# Communicating information about “what not to do” to consumers

**DOI:** 10.1186/1472-6947-13-S3-S2

**Published:** 2013-12-06

**Authors:** John S Santa

**Affiliations:** 1 Consumer Reports Health Ratings Center, 101 Truman Avenue, Yonkers, NY – 10703, USA

## Abstract

**Background:**

Americans devote more resources to health care than any other developed country, and yet they have worse health outcomes and less access. This creates a perfect set of opportunities for *Consumer Reports*, a nonprofit consumer advocacy and multimedia organization known for its focus on individual consumers in markets where significant amounts of misinformation is in play. *Consumer Reports* uses comparisons/ratings based on the best available data to “level” the market playing field. While our early efforts to inform consumers of overuse and underuse in health care were successful, we sensed there were opportunities to have greater impact. Over a 5-year period, with the help of many partners, *Consumer Reports* learned more about how to communicate “what not to do” to consumers, ultimately enhancing the effectiveness of this difficult message.

**Analysis:**

*Consumer Reports* began an in-depth examination of the overuse of health services in 2010 with an exploratory review of the cognitive psychology literature. Lessons learned from this review were used in the presentation of subsequent ratings of heart disease and cancer screening tests. Surveys showed surprising gaps in the prevention/screening knowledge of healthy, low-risk *Consumer Reports* subscribers aged 40 to 60 years. Of these subscribers, 44% reported engaging in heart screening tests that received the lowest ratings from the U.S. Preventive Services Task Force and from *Consumer Reports.* At the same time professional societies and the ABIM (American Board of Internal Medicine) Foundation created Choosing Wisely^®^, a campaign focused on identifying lists of health tests and treatments not to do. *Consumer Reports* joined the Choosing Wisely campaign as the consumer “translator” and organizer of a network of consumer organizations with access to tens of millions of consumers. Over the past year, *Consumer Reports* has conducted multiple qualitative evaluations of content related to overuse of health services. Ratings of cancer screening tests were released in 2013 in an article readers reported as one of the most heavily read articles in the magazine’s recent history.

**Conclusions:**

Telling consumers that more is not better is not a popular or easy message to deliver. The message is most likely to be “sticky” but is best received if it comes from trusted sources (e.g., physicians), focuses on safety when justified, is communicated in plain language, and uses both mass media and individual consumer approaches. Changing the culture of health care in an era of health reform is an essential part of the transformation needed if we are to allocate finite resources fairly in hopefully fair markets while assuring that better quality products and services at lower prices dominate.

## Background

Seventy-seven years ago, consumer activists—some might call them radicals—foresaw a future in which consumers would be repeatedly put at a disadvantage in American markets by advertising and promotion. Their efforts led to the founding of *Consumer Reports*, a publication focused on a simple but elegant solution—independent comparisons or “ratings” especially focused on highly advertised products and services. The first edition of *Consumer Reports* in 1936 included a review of Alka-Seltzer, with findings that questioned the preparation’s effectiveness. It was, after all, aspirin with a fizz following a plop in a glass of water.

Since that time, American markets have provided the world with multiple examples of productive competition but also of failed markets in which costly, inefficient, or unsafe products or services prevailed. *Consumer Reports* has become an icon to individual consumers who need help to improve their odds of economic, and at times physical, survival. Advertising and promotion focuses on benefits, usually with only required disclosure of risks. While *Consumer Reports* frequently covered health-care markets, it was not until 2007 that the organization elevated health care to a “franchise” within the company that is similar to producers of cars, electronics, and household goods.

This focus on health care was only fitting, given the rising portion of the wealth of the United States devoted to health and the increasing frequency of sophisticated promotional techniques used by pharmaceutical companies, hospitals, health insurers, and doctors. But health care is a unique industry. The primary industry connection to the customer is the doctor whose professional code should prioritize the best clinical outcomes for the patient and include a fiduciary responsibility.

*Consumer Reports* was not the first organization to appreciate the many shortcomings in the health-care market. But its unique assets are formidable—independence from industry, commitment to the best data possible, iconic presentation skills, and a distribution network of tens of millions of savvy consumers. But the organization’s most powerful tool is the acceptance—if not the expectation—that *Consumer Reports* will communicate to consumers about both good performers and poor performers, about benefits and risks, and about underuse and overuse of health services.

In 2007, in partnership with the BMJ Group, *Consumer Reports* published ratings of treatments for more than 200 conditions based on evidence from Cochrane reviews [1]. In 2008, ratings of chronic care services by hospital, which were based on the *Dartmouth Atlas of Health Care*, followed [2]. These comparisons revealed variations in health services that were well known to the medical community but were a surprise to many consumers. Further work, especially around hospital and prescription drug comparisons, countered common advertising and promotional messages from industry. But it also became apparent that messaging around risk and overuse, especially in a sea of “more is always better” messages, was challenging. How then do we tell consumers what not to do?

## Analysis

### Review of cognitive psychology literature

In the fall of 2010, *Consumer Reports*, with funding from the Agency of Healthcare Research and Quality, conducted a review of the cognitive psychology literature [3]. As stated in the review, “The goal was to compile lessons learned from cognitive science research about how people process information in order to consider how best to communicate with consumers about those specific preventive health services that are not recommended for most.”

The review was exploratory and selective in nature, developing “several contextual variables and guiding principles that might facilitate communication about preventive health services people should not do.” The results were crucial in eventual work on overuse of health services and shaped our strategies.

#### **1. People tend to continue acting in ways they have acted in the past.**

Americans are well experienced in the use of a health-care system that usually advises more is better and more expensive is better. They also are more likely to consult information sources they have used in the past when it comes to health care. Building a following for an information source focused on overuse would be challenging.

#### **2. Focusing people’s attention on different aspects of the same information (via message framing) can alter people’s ultimate decisions. People tend to choose positively described options when they perceive options as safe, and people tend to choose negatively described options when they perceive options as risky.**

Our focus turned to established communication outlets that were engaged in health discussions or topics that could be reasonably connected to a health topic. We have explored message framing approaches that attract people’s interest in overuse. Safety/risk themes are especially effective. Comparisons are able to capture both benefits and risk in symmetrical ways that attract readers.

#### **3. People process information both analytically and experientially, and as such the emotional content of messages must be considered.**

Stories that capture the message are important, especially if they come from a storyteller with whom the reader identifies. Messaging around benefits has used such stories for decades, generating controversies about the applicability and fairness of the anecdote.

#### **4. Decision aids may improve decision making for preventive health options that are not recommended.**

Messaging around overuse “breaks” the consumer’s expectations about many health issues. A credible approach will “fix” this break. However, the consumer may be wary of the fix and look for a variety of reassuring factors. Messaging that tells the consumer to not do what they would usually do also needs to tell them what to do.

### Comparisons of screening tests

During this same time frame, *Consumer Reports* began a process to compare and rate screening tests for heart disease. The process began with access to the best data available—systematic reviews done for the U.S. Preventive Services Task Force (USPSTF). An internal review group of *Consumer Reports* staff reviewed the evidence with the help of external consultants active in the field. The ratings methodology included a search for evidence published subsequent to the USPSTF recommendation, an estimation of the burden of disease each screening test would address, the costs associated with each screening test (including downstream costs of additional testing and subsequent treatment), and finally any collateral benefit the screening tests might have for diseases other than cardiac disease. A series of ratings tables were generated online and in *Consumer Reports* magazine. The ratings tables had a *Consumer Reports* “look,” as in Table 1[4].

**Table 1:** Consumer Reports heart/vascular prevention test ratings: asymptomatic men, 45–54 years of age

Heart test	Rating	Benefits	Risks	Cost
Blood pressure		Substantial	Minimal	Minimal
Cholesterol		Substantial	Minimal	Minimal
Blood glucose (diabetes)		Minimal	Minimal	Minimal
C-reactive protein		Minimal	Minimal	Minimal
Clogged peripheral arteries		Minimal	Moderate	Substantial
Clogged carotid arteries		None	Moderate	Substantial
Abdominal aortic aneurysm		None	Moderate	Substantial
Electrocardiogram (EKG or ECG)		None	Moderate	Moderate
Stress test (EKG)		None	Moderate	Moderate

Footnote: Ratings focus on whether “benefits outweigh risk.” The symbol key is as follows:

Very Likely  Likely  Uncertain  Unlikely  Very Unlikely

The process also included a survey of consumers around their experiences with heart disease screening tests [5]. The results were surprising. The findings from a survey of a subset of 1266 healthy, asymptomatic, low–risk, 40- to 60-year-old subscribers to *Consumer Reports* (not a nationally representative sample) were especially informative; 44% had had a low-rated (according to the USPSTF and *Consumer Reports*) cardiovascular screening test. The most common, low-rated screening test was an electrocardiogram (EKG), followed by a stress test and ultrasound. The respondents significantly overestimated their risk for cardiovascular disease.

The survey also asked respondents’ knowledge of screening tests based on physician communication. Given that subscribers of *Consumer Reports* are more likely to be highly educated and affluent, the results were again surprising [6]: 11% had talked with their physician about follow up if the screening test was abnormal; 9% had discussed the accuracy of the test; 4% knew about the potential complications from the test; and 1% had discussed with their physician whether or not the test saved lives [7].

In September 2011, *Consumer Reports* published a feature story in its magazine focused on heart disease that included an investigative story on angioplasty, a summary of the heart screening test ratings, and ratings of heart surgeon group performance. The 2011 feature story and derivative reports in other publications employed many of the communication approaches identified as effective earlier [3]. The story was internally competitive—the cover was devoted to the story and the ratings. Readership rates were high, especially for the information about screening tests, and media coverage and the subsequent interest of industry were robust. However, this experience also had its challenges. Consumer feedback on many of the screening topics expressed wariness: “How could a simple test like EKG, one that I recall receiving routinely many times, not be good, even be unsafe?” *Consumer Reports* heard from individual consumers their anecdotes about reactions from physicians that ranged from surrender to full-on attack.

### Choosing Wisely^®^

In February 2011, the American College of Physicians (ACP) announced their commitment to an effort focused on “high value, cost conscious care” [8]. In the spring of 2011, *Consumer Reports* and the ACP began discussions around communication with consumers concerning overuse of coronary disease screening services. In a subsequent January 2012 publication, the ACP announced 37 “Clinical Situations in Which a Test Does Not Reflect High-Value Care” [9]. The list included several screening tests for heart disease, such as EKG and stress tests, in low-risk patients [9].

During the same period, the ABIM Foundation had expanded work on the Physician Charter [10], emphasizing approaches to the allocation of finite resources. Howard Brody’s perspective, published in the *New England Journal of Medicine* in January 2010, called for physician groups to identify the five most “egregious” causes of waste [11]. With support from ABIM Foundation, the National Physician Alliance published a “Top 5 list” of “activities…where the quality of care could be improved” in *Internal Medicine*, *Family Practice*, and *Pediatrics *[12].

By the fall of 2011, eight other professional societies had joined the ACP in committing to the development of a Top 5 list. Professional societies developed their own process but were required to meet four requirements: (1) items in each list needed to be within the purview and control of the specialty represented; (2) the clinical procedures selected for inclusion in the lists should be used frequently and/or carry a significant cost; (3) and there should be generally accepted evidence to support each recommendation; (4) and the process should be thoroughly documented and publicly available upon request. Harmonization across societies was not required but desirable. The ABIM Foundation organized an effort to support the Top 5 listing process, including a communications campaign and outreach to additional professional societies. The objective of the campaign was to initiate conversations between patients and physicians about overuse [13].

*Consumer Reports* joined the campaign to assist in the consumer-oriented portion of the campaign. With the results of the previous cognitive psychology review in mind, *Consumer Reports* focused its resources on two objectives:

1. Translation of Top 5 lists in partnership with each professional society through use of a shared, mutual consent editorial process. The objective of these documents was to explain each topic in plain language and offer consumers advice about what they should do and not do—all under the endorsement of two trusted brands—the professional society and *Consumer Reports *[14].

2. Organization of a coalition of consumer communication partners to disseminate content and messages about the appropriate use of the medical procedures to the communities they serve. Consumer partners committed to the distribution of Choosing Wisely materials to at least 1 million consumers. These partners included AARP (formerly the American Association of Retired Persons), several business coalitions, labor organizations, Wikipedia, Univision, the National Center for Farmworker Health, and Alliance Health Networks.

Choosing Wisely was announced in April 2012. Coverage in the consumer and trade press was substantial and sustained, eventually reaching an estimated 300 million media impressions. Choosing Wisely was also eventually mentioned in more than 100 medical journal articles. By February 2013, *Consumer Reports* estimated that 80 million consumers had seen content about one or more topics via the consumer network.

In February 2013, a second wave of 17 professional societies announced 90 Top 5 topics. The topics went well beyond diagnostic testing, with many focusing on screening, treatment, preoperative evaluation, and routine monitoring. Participating societies included the American Academy of Pediatrics, the American College of Obstetrics and Gynecology, and the American Geriatric Society—creating topics from birth to end of life [15].

Wikipedia launched a *Choosing Wisely* page [16], and three employer coalitions launched a tool kit to enable improved access to *Choosing Wisely* content [17]. Univision joined the consumer network, committing to publication of Spanish translations of *Choosing Wisely* content on a dedicated Web page. In addition, Leapfrog published updated performance measurements of hundreds of hospitals’ early elective delivery rates—on the same day that the American Academy of Family Physicians and ACOG announced the selection of elective delivery before 39 weeks as a Top 5 topic to be addressed through Choosing Wisely.

### Qualitative studies of overuse communication

The success of Choosing Wisely and other overuse content in *Consumer Reports* prompted multiple calls for measurement of its effects. The ABIM Foundation and *Consumer Reports* met with researchers in the summer of 2012 to discuss the feasibility of such measurement. The conclusion from the session was that quantitative measurement of most, if not all, of the topics identified at that time would be difficult and attribution to Choosing Wisely or *Consumer Reports* efforts would be even more challenging because many of the topics were also being pursued by other industry groups. Imaging test overuse, for example, was already a common topic among health insurers, and radiologists had launched campaigns—Image Wisely and Image Gently—to moderate imaging [18].

Before the April 2012 announcement, both the ABIM Foundation and *Consumer Reports* had conducted market research about messaging related to overuse. *Consumer Reports* conducted focus groups around messaging related to specific topics such as EKG screening. The results of these focus groups emphasized the wariness of consumers around overuse messaging, the importance of physician agreement and concerns that such messaging might be related to health insurer or government efforts.

The ABIM Foundation had engaged Michael Perry and Tressa Undem, formerly of Lake Research Partners, from 2009 to 2011 to conduct both focus groups and a survey of physicians to explore concepts around the “just distribution of finite resources” as articulated in the Physician Charter. Ultimately, the ABIM Foundation concluded from this research that “when physicians were presented with language that moved away from the interests of the patient or the well-being of physicians, and toward community and the need for a sustainable health system, they were less motivated to take action”. The physicians agreed that phases such as “wise choices” accurately reflected their desire to empower their patients to make informed decisions about their treatment, while encompassing the ideals of the *Physician Charter* they sought to live up to [19].

*Consumer Reports* focused on a series of descriptive or qualitative studies to help evaluate the translation work. Several approaches were pursued:

• The National Center for Farmworker Health conducted a survey of clinicians and staff working in multiple clinics across the country asking them to evaluate the two-page *Choosing Wisely* topic translations. Participants advised that the content of the two-page consumer summaries were: recognizable, credible, and valuable, except for the physician association brands, which were less recognizable; the text could be difficult to understand for the average patient, whether in English or Spanish; and some images were hard for patients to identify with but they were helpful in communicating overuse, even to low-income patients [20].

• Health Research for Action at the University of California Berkeley conducted a series of one-on-one interviews as they translated topic summaries into plain-language versions [21]. The interviewees noted that:

○ Although trust is important, brands are not recognizable in many cases.

○ Advice on what to do is the most popular part of the content.

○ Self-care, nondrug, and nonphysician advice is the most popular type of advice about what to do.

○ Text can be intimidating; bullets are more effective.

○ Pictures are important to set the tone, for consistency and for conveying the message.

○ Although health information is considered credible, the preference is to talk with a doctor and do what he or she says.

○ Brand names are much more recognizable; generic names are confusing.

• The Consumer Insight Group at *Consumer Reports* (market research team) conducted a survey of 2,269 nationally representative individuals to evaluate the two-page Choosing Wisely pamphlets. Based on the feedback received, the pamphlets are now written in plain language, use more consumer-friendly formats and pictures, and contain significant self-care advice. The responses were largely positive, with 75% to 85% of readers interested in the topic being extremely likely or somewhat likely to talk with their doctors about the topics based on what they read [22].

In February 2013, the ABIM Foundation, *Consumer Reports*, and the Robert Wood Johnson Foundation announced a grant to the ABIM Foundation and *Consumer Reports* that is designed to better implement Choosing Wisely in physician and consumer communities across the United States. An evaluation of the outcome of these efforts is included in the proposed research [23].

### Ratings of cancer screening tests

In January 2013, *Consumer Reports* released ratings of 11 cancer screening tests in low-risk, asymptomatic people; eight tests received low ratings. The story and ratings were featured on the cover of *Consumer Reports.* Many of the lessons learned from previous efforts were integrated into this release. The story began with an anecdote about a physician who had pursued a mildly elevated prostate-specific antigen test only to become septic after a prostate biopsy (an empathic story with a safety storyline). It also included a summary of ratings for a decision aid focused on low-risk individuals and featured three cancer screening tests that were effective (attracting people interested in benefits) and information for individuals at high risk for the types of cancer detected by the tests—telling readers what to do and what not do.

The ratings attracted substantial media attention, including coverage on a national morning news show and on The Dr. Oz Show. We highlighted the content of the article in which people acknowledged that they would be less likely to look for information about overuse.

Readership rates for the article on cancer screening test ratings were high and were similar to those of the most-read articles in the recent history *Consumer Reports *[24]. The story will be distributed free of charge to consumer audiences through the same distribution network used by Choosing Wisely.

## Conclusions

Taking on communication about health services overuse to consumers is tricky because American culture, including direct-to-consumer advertising, promotes demand. Messaging that promotes “more is better” and “more expensive is better” is difficult to counter. However, this difficulty may be overcome, especially by an organization whose subscribers expect them to do just that. The message is most likely to be “sticky” and well understood if it comes from trusted sources that include physicians, focuses on safety when appropriate, is communicated in plain language, and uses both mass media and individual consumer approaches. Approaches to communicating about overuse via mass media can work well if the message is “right” and large organizations with access to consumers endorse the message. Changing the culture of health care in an era of health reform is an essential part of the transformation needed if we are to allocate finite resources fairly in hopefully functional markets—better quality products and services at lower prices should dominate.

The experience with Choosing Wisely indicates that strategies can be developed and deployed successfully in heightening consumer understanding of the benefits and harms associated with specific medical procedures and services. Further development, testing, and documentation of how public communication strategies and campaigns can contribute to wiser allocation of finite resources to health services that produce measurable benefits should be an important focus of public and private efforts going forward.

## Abbreviations used

AARP: American Association of Retired Persons; ABIM: American Board of Internal Medicine; ACOG: American College of Obstetrics and Gynecology; EKG: electrocardiogram; USPSTF: U.S. Preventive Services Task Force

## Competing interests

The author is employed full time by Consumer Reports Health as Director of the Health Ratings Center. As a condition of employment no other conflicting financial or non financial relationships are allowed.

## Declarations and disclaimer

The Eisenberg Conference Series 2012, *Supporting Informed Decision Making When Clinical Evidence and Conventional Wisdom Collide*, was conducted in Rockville, Maryland, by the John M. Eisenberg Center for Clinical Decisions and Communications Science, Baylor College of Medicine, Houston, Texas, for the Agency for Healthcare Research and Quality under Contract No. HHSA 290-2008-10015-C. Publication costs for this supplement were funded by this contract. The author of this article is responsible for its content. No statement may be construed as the official position of the Agency for Healthcare Research and Quality and of the U.S. Department of Health and Human Services.

This article has been published as part of *BMC Medical Informatics and Decision Making* Volume 13 Supplement 3, 2013: Articles from the Eisenberg Center Conference Series 2012: Supporting informed decision making when clinical evidence and conventional wisdom collide. The full contents of the supplement are available online at http://www.biomedcentral.com/bmcmedinformdecismak/supplements/13/S3.
